# Broad-spectrum therapeutic potential of 4-phenylbutyrate in neurological and systemic diseases of viral and non-viral origin

**DOI:** 10.3389/fphar.2025.1621590

**Published:** 2025-08-21

**Authors:** Fatima Bobat, David Wu, Ethan Tu, Divya Kapoor, Pankaj Sharma, Joseph S. Adams, Chima Orameh, Tejabhiram Yadavalli, Abhijit Date, Deepak Shukla

**Affiliations:** ^1^ Department of Ophthalmology and Visual Sciences, University of Illinois Chicago, Chicago, IL, United States; ^2^ University of Illinois Urbana-Champaign, Champaign, IL, United States; ^3^ Department of Microbiology and Immunology, University of Illinois Chicago, Chicago, IL, United States; ^4^ The University of Arizona, Tucson, AZ, United States

**Keywords:** 4-phenylbutyrate (4-PBA), herpes simplex virus, neurological disorders, Broadspectrum, therapeutic

## Abstract

4-Phenylbutyrate (4-PBA), initially recognized for treating urea cycle disorders, has emerged as a potent therapeutic agent with broad-spectrum potential. As a chemical chaperone, 4-PBA modulates protein folding and reduces endoplasmic reticulum stress. 4-PBA has demonstrated efficacy in treating ocular herpes simplex virus type 1 (HSV-1) infection and HSV-1-induced encephalitis, highlighting its potential as a novel anti-herpetic therapy. Beyond its antiviral properties, 4-PBA’s therapeutic reach extends to neurological disorders linked to HSV-1 infection, including Parkinson’s, Alzheimer’s, Huntington’s diseases, and primary open-angle glaucoma. Furthermore, 4-PBA shows promise in treating a diverse array of conditions beyond neurology. Its potential has been explored in atherosclerosis, Adriamycin-induced cardiac injury, non-alcoholic fatty liver disease, rifamycin-induced liver injury, chronic kidney disease, diabetic nephropathy, NSAID-induced kidney injury, and chronic wound healing. This review synthesizes the multifaceted therapeutic potential of 4-PBA, emphasizing its role as a broad-spectrum agent capable of addressing a wide range of pathological conditions, particularly its role in combating HSV-1 and associated neurological disorders. The growing evidence suggests that 4-PBA may be a versatile and valuable addition to the therapeutic arsenal against multiple diseases.

## 1 Introduction

The global burden of disease, encompassing a wide range of chronic and infectious conditions, poses substantial humanitarian and economic challenges, necessitating continuous evaluation and adaptation of healthcare strategies. In the pursuit of novel and effective therapeutic approaches, the pharmaceutical landscape has witnessed a surge in the exploration of small-molecule compounds with multifaceted applications. Among these, the chaperone molecule 4-phenylbutyrate (4-PBA) has emerged as a compelling agent with the potential to treat a diverse spectrum of medical conditions. Originally investigated for its role in relieving endoplasmic reticulum (ER) stress in genetic disorders, recent research has unveiled the expansive therapeutic implications of 4-PBA across a spectrum of organ systems (Yam et al., 2007; Takatori et al., 2017; Wang et al., 2018; Bonnemaison et al., 2019; Paganoni et al., 2020; Maddineni et al., 2021). These studies outline the potential of the drug to subdue the burden of disease worldwide. However, there currently lacks a consolidated body of information on 4-PBA. There is a significant need for a body of work that aggregates our current understanding of this drug from a basic science, pharmacologic, and clinical perspective. This review poses to address that knowledge gap by outlining the therapeutic applications of 4-PBA to a variety of organs in the cardiovascular, hepatic, renal, neurologic, ocular, and integumentary systems. It will also highlight the mechanism of action for 4-PBA on its targets within each system and provide insights into future research on this agent.

## 2 Methods

Based on their known involvement with inflammatory responses, endoplasmic reticulum (ER) stress, and protein misfolding, 13 different diseases across 6 major organ systems were selected for review: cardiovascular, hepatic, renal, neurological, ocular, and integumentary. To evaluate the systemic applications of 4-PBA, a comprehensive literature review was conducted using the databases Scopus, PubMed, and Web of Science to search for keywords “4-PBA” and “ER stress,” “unfolded protein response,” or “chemical chaperone,” and each specific disease name. Experimental *in vitro* and *in vivo* models were included as well as preclinical and clinical models, prioritizing studies that assessed the mechanism of action, safety profile, and pharmacologic efficiency.

## 3 Background of phenylbutyrate

**FIGURE 1:**
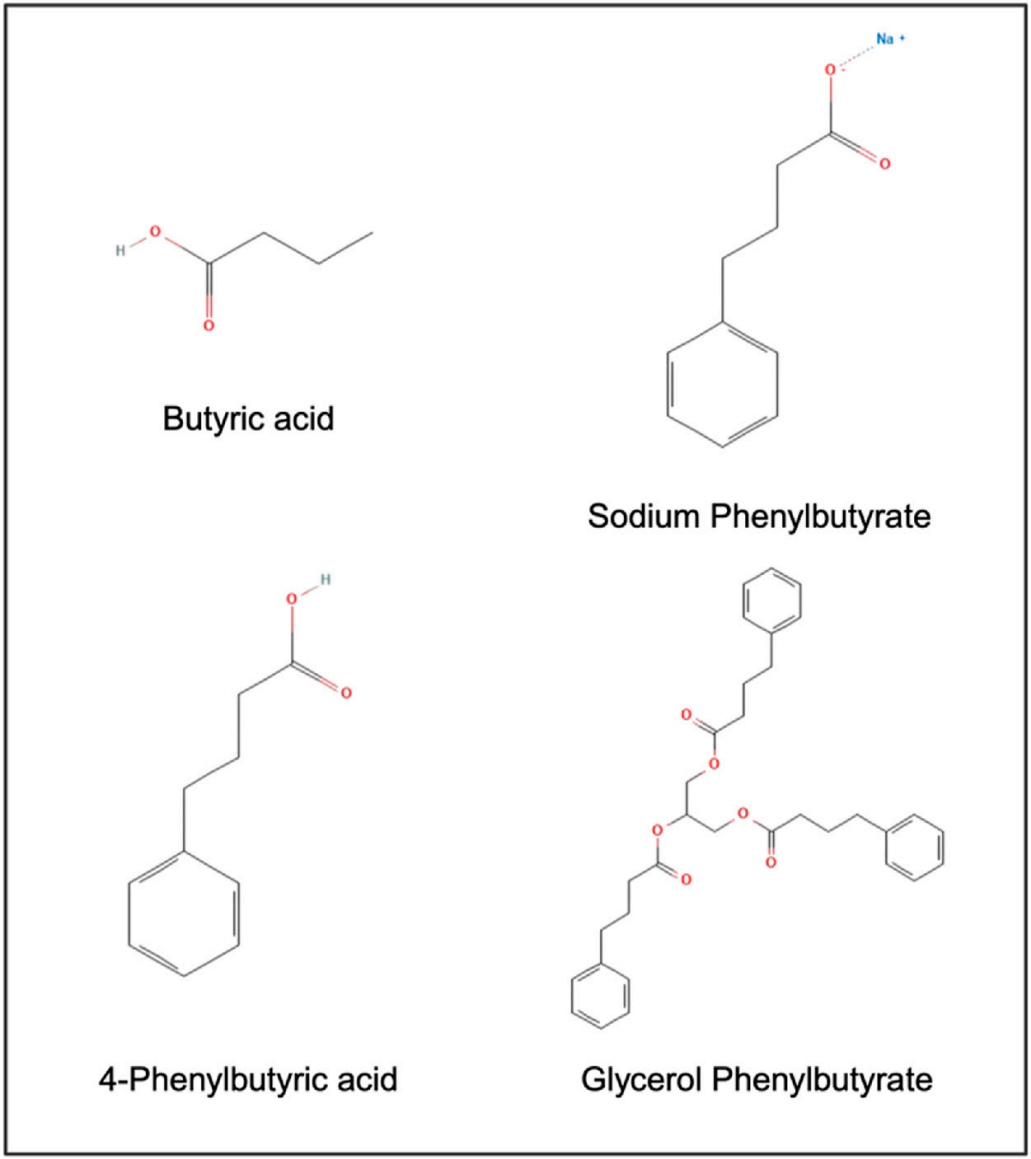
Chemical structure of butyric acid, 4-phenylbutyric acid (PBA), and PBA prodrugs.

Phenylbutyric acid (PBA) also known as 4-Phenylbutyrate (4-PBA) is a short chain aromatic fatty acid with a phenyl ring attached to a 4-carbon chain with a carboxylic acid terminating group. For pharmaceutical applications, 4-PBA is available as a sodium salt and in the form of a prodrug, glycerol phenylbutyrate (Figure 1).

### 3.1 Discovery of phenylbutyrate

**FIGURE 2:**
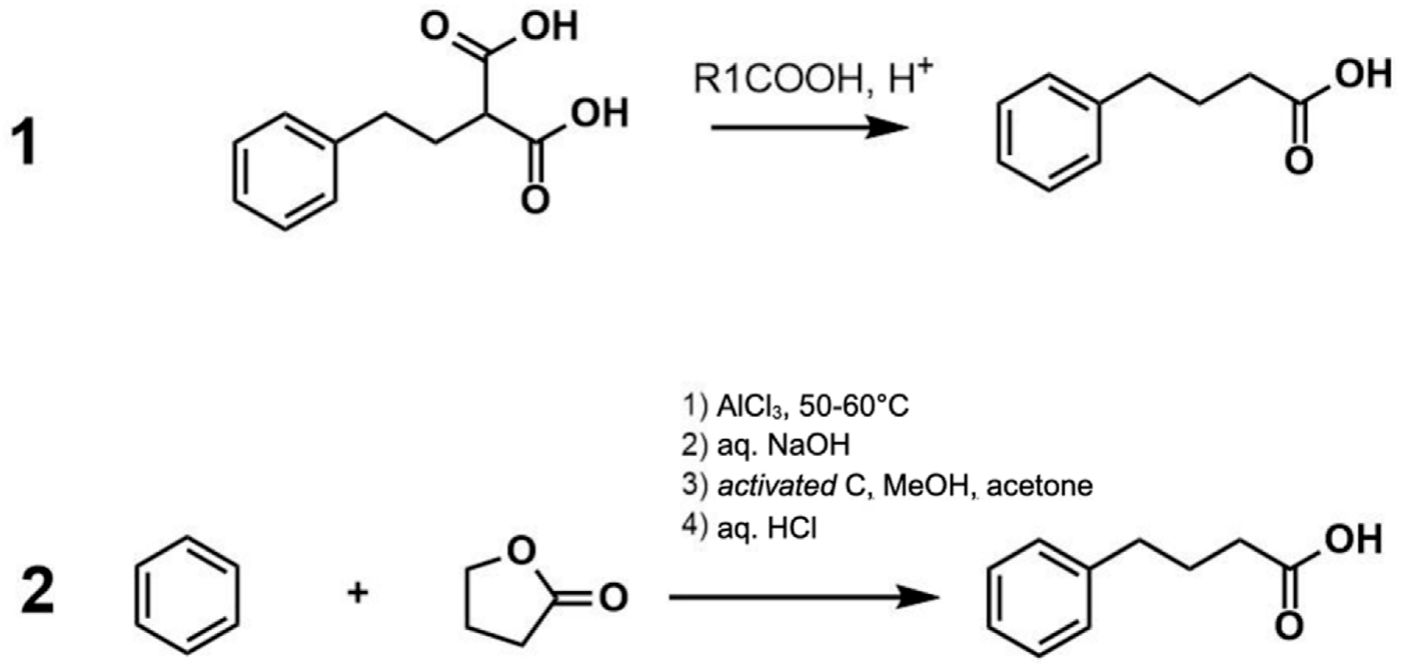
PBA synthesis methods 1) Jobdevairakkam and Muthiah (2007) US 2007/0004805 A1 2) Burzynski and Musial (2002) US 6,372,938 B1.

4-PBA was first described by Franz Knoop in 1904 during the discovery of the process of fatty acid oxidation. In this investigation, the administration of 4-PBA to dogs led to the generation of phenylacetylglutamine (Houten and Wanders, 2010). As the exploration of the physiological and therapeutic roles of endogenously generated phenyl carboxylic acids continued, phenylacetic acid (PAA), an endogenous metabolite of the amino acid phenylalanine, showed great potential for the treatment of hyperammonemia in children and adults and several other ailments (Nagamani et al., 2018). However, the clinical potential of PAA was severely limited due to its highly unacceptable odor, likened to a horse stable, which remained in the formulations as well as in the patients after ingestion. Interestingly, as a part of the fatty acid oxidation process discovery, it was already known that 4-PBA is metabolized to PAA, which eventually is converted to phenylacetylglutamine. Furthermore, 4-PBA did not have an unacceptable odor, unlike PAA, and was available in solid form, making it a promising therapeutic candidate in the early years (Newmark and Young, 1995). Hence, 4-PBA gained interest first as a precursor of PAA for the treatment of hyperammonemia, and eventually several other therapeutic roles of 4-PBA, such as histone deacetylase (HDAC) inhibitor and neuroprotectant, were uncovered (Iannitti and Palmieri, 2011). Since then, 4-PBA has been approved by the FDA for the treatment of urea cycle disorders (UCD) in 1996 and more recently for the treatment of amyotrophic lateral sclerosis (ALS). The industrial synthesis of 4-PBA is relatively simple, and several methods have been patented and since abandoned, with two of the best described synthesis methods described in Figure 2. Most commonly, it is produced by reacting benzene with butyrolactone via an aluminum chloride catalyst. The resultant product is then neutralized with a base to produce sodium phenylbutyrate (sPBA) (Figure 2).

### 3.2 Breakthrough use of 4-PBA in treating urea cycle disorders

**FIGURE 3:**
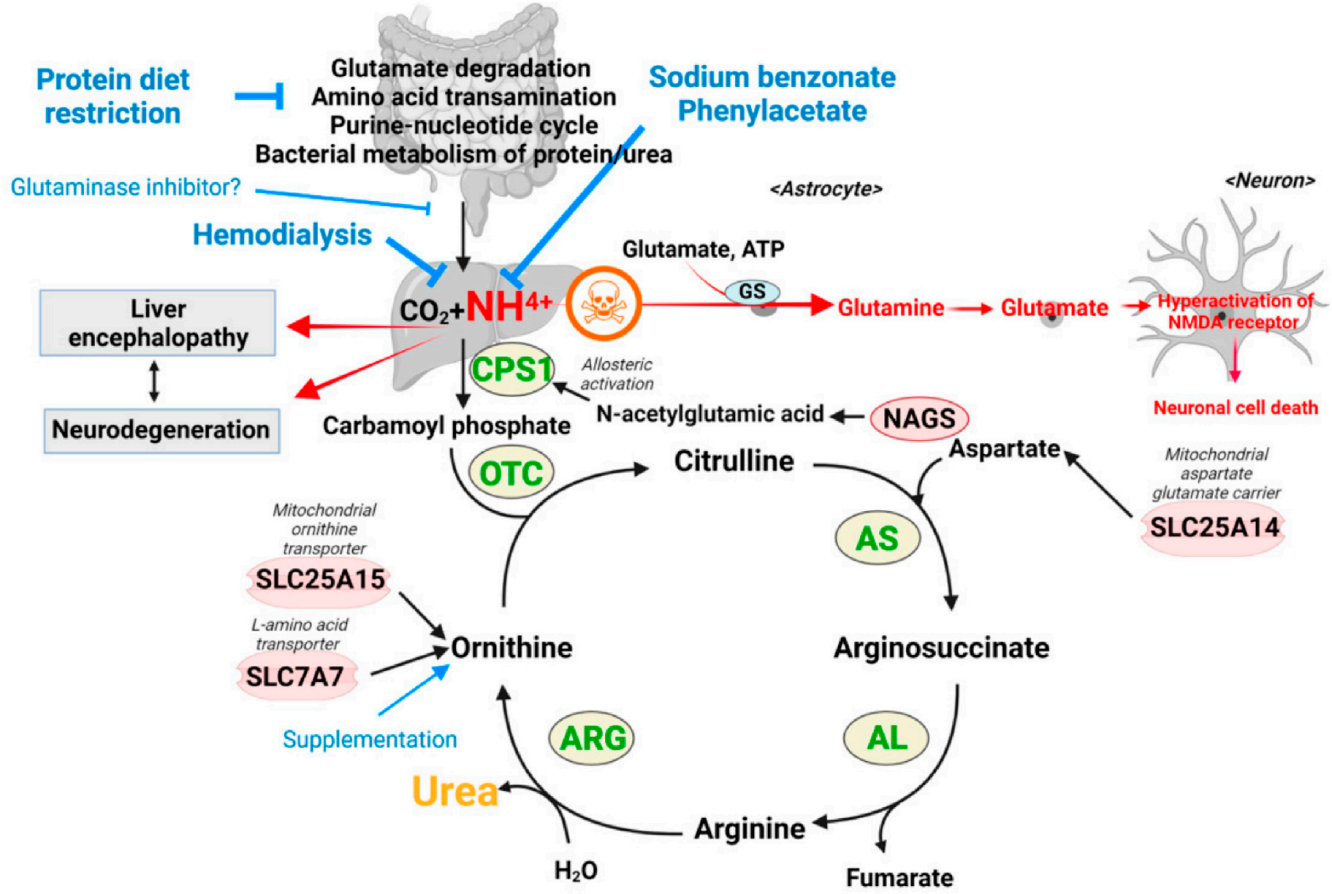
Urea cycle disorder - reproduced from (Lee and Kim, 2022).

The first therapeutic use of 4-phenylbutyric acid (4-PBA) was for the treatment of urea cycle disorders (UCDs), a cluster of rare diseases that affect the body’s ability to metabolize and excrete nitrogenous waste (Figure 3). The most common UCDs include CPS1 deficiency, OTC deficiency, and the citrullinemias, all resulting in the buildup of ammonia (Summar and Mew, 2018). These diseases result in a constellation of symptoms including lethargy, vomiting, poor feeding, and, in grave cases, permanent neurotoxic damage (Lopes et al., 2022). There are many commercialized formulations of 4-PBA, as it is now a generic license compound. Notably, these products are exclusively licensed for urea cycle disorders rather than any other indication discussed in this review. It is currently available as an intravenous bolus, tablet, powder, and suspension. Table 1 represents the currently available 4-PBA products, Table 2 represents the diseases possibly treated by 4-PBA, and Table 3 provides the list of cliunical trials for 4- PBA.

**TABLE 1:** Currently available PBA products–adapted from (Häberle et al., 2012).

Compound	Brand	Form
Sodium Benzoate and sodium PBA	Ammonul (Valeant Pharmaceuticals)	IV
Sodium PBA	Pharma international	IV
Sodium PBA	Ammonaps (Swedish Orphan Biovitrum)	Oral
Sodium PBA	Buphenyl (Horizon Pharma)	Oral
Sodium PBA	Buphenyl (Horizon Pharma)	Powder
Sodium PBA	Pheburane (Lucane Pharma)	Granules
Glycerol PBA	Ravicti (Horizon Pharma)	Granules

**TABLE 2:** List of diseases possibly treated by 4-PBA.

S.No.	Disease	Mechanism of action	References
1	Cystic Fibrosis	As a potential therapy, it acts as chemical chaperone and assists in proper protein folding to improve CFTR in affected individuals	Lim et al. (2004)
2	Urea Cycle Disorders	It helps in the treatment of urea cycle disorders by decreasing ammonia levels in the body. It does so by enhancing the excretion of nitrogenous waste products in the form of phenylacetylglutamine	Lee et al. (2010)
3	Familial Amyloid Polyneuropathy (FAP)	4-PBA can potentially slow down the progression of the disease by stabilizing effect on the misfolded transthyretin (TTR) protein aggregates in tissues that causes the damage in FAP	Valastyan and Lindquist (2014)
4	Niemann-Pick Disease Type C (NPC)	NPC is characterized by defective cholesterol trafficking within cells. 4-PBA has been explored for its ability to correct cholesterol homeostasis in NPC by promoting the expression of NPC1 protein and enhancing its stability. This helps in improving cellular cholesterol transport	Kim et al. (2007)
5	Alzheimer’s Disease	In Alzheimer’s, misfolded proteins such as beta-amyloid accumulate, leading to neurodegeneration. 4-PBA’s chaperone-like effect may assist in preventing misfolding and aggregation of these proteins. Additionally, it has been suggested to have anti-inflammatory and neuroprotective properties	Ricobaraza et al. (2009)
6	Parkinson’s Disease	The exact mechanism of 4-PBA in Parkinson’s Disease is not fully elucidated, but it’s thought to exert neuroprotective effects. It may help in reducing oxidative stress, inflammation, and enhancing cellular resilience, which could be beneficial for dopaminergic neurons	Tiwari et al. (2022)
7	Huntington’s Disease	Huntington’s is characterized by the accumulation of misfolded huntingtin protein. 4-PBA’s chaperone-like properties may aid in the proper folding of huntingtin, potentially reducing its toxic effects on cells	Alfahel et al. (2022)

**TABLE 3:** List of clinical trials for 4-PBA (https://clinicaltrials.gov/).

S.No.	Disease	Identification number	Status	Purpose
1	Alpha 1-Antitrypsin Deficiency	NCT00067756	Completed (2003-10)	The purpose of this study is to determine whether 4-PBA will significantly increase serum Z AAT levels in AAT-deficient individuals with and without evidence of hepatocellular injury and to assess its effects on liver injury
2	Immune Reconstitution in HIV Disease (IREHIV)	NCT01702974	Completed (2015-08)	The aim with this study is to provide immunotherapy with vitamin D and phenylbutyrate to treatment-naive HIV infected patients to induce important antimicrobial defense mechanisms and decreased inflammation
3	Immune Reconstitution in Tuberculosis Disease (IRETB)	NCT01702974	Completed (2015-08)	The aim with study is to provide adjunctive therapy with vitamin D and phenylbutyrate together with standard anti-tuberculosis treatment to significantly improve clinical recovery among patients with untreated, active pulmonary tuberculosis
4	Urea Cycle Disorders	NCT02111200	Completed (2015-10)	The investigators studied and compared how effectively sodium phenylbutyrate, sodium benzoate, and a combination of the two, help excrete nitrogen in healthy volunteers
5	Medium Chain Acyl-CoA Dehydrogenase Deficiency (MCADD)	NCT06069375	Recruiting	This is a medical research study to test a medication in patients 10 years of age and older with a disease called medium-chain acyl-CoA dehydrogenase deficiency (MCADD) caused by the common ACADM c.985 A>G (K304E) mutation
6	Spinal Muscular Atrophy (STOPSMA)	NCT00528268	Completed (2013-12)	In this single-center trial, we will evaluate the effects of NaPB on presymptomatic Spinal Muscular Atrophy (SMA) type I (cohort 1) and presymptomatic SMA type II (cohort 2) infants
7	ALS (Amyotrophic Lateral Sclerosis)	NCT00107770	Completed (2007-09)	The purpose of the study is to evaluate the safety of sodium phenylbutyrate (NaPB) treatment in subjects with amyotrophic lateral sclerosis (ALS) and the ability to take this medication without major side effects
8	Spinocerebellar Ataxia Type 3	NCT01096095	Withdrawn	This trial aimed to assess the safety and efficacy of 4-PBA in patients with ALS.
9	Wolfram Syndrome	NCT05676034	Recruiting	AMX0035 (Proprietary formulation of taurursodiol and sodium phenylbutyrate) is a combination therapy designed to reduce neuronal death through blockade of key cellular death pathways originating in the mitochondria and endoplasmic reticulum (ER). This clinical trial is an open label Phase II study to evaluate the safety and efficacy of AMX0035 in adults with Wolfram syndrome
10	MCT Mutation (Allan-Herndon- Dudley Syndrome)	NCT05019417	Unknown	They hypothesized that treatment of AHDS patients with glycerol phenylbutyrate (GPB) will improve thyroid function and neurodevelopmental parameters and relieve symptoms resulting from toxic T3 levels in peripheral tissues. Objective: To test safety and efficacy of PB treatment in AHDS patients

4-PBA’s first discovered medical use and subsequent pharmaceutical approval by the FDA in 1996 was for the excretion of excess urea in patients with urea cycle disorders (Peña-Quintana et al., 2017). Urea cycle disorders (UCDs) arise from genetic mutations that cause a deficiency or malfunction in one of the critical enzymes involved in the urea cycle. These enzymes include carbamoyl phosphate synthetase, argininosuccinic acid lyase, N-acetylglutamate synthase, ornithine transcarbamylase, and argininosuccinic acid synthetase. Urea cycle disorders are a cluster of disorders that affect the urea cycle and ultimately lead to a buildup of nitrogenous waste. For instance, a deficiency of carbamoyl phosphate synthetase 1 (CPS1) results in CPS1 deficiency. The absence or dysfunction of these enzymes is a rare genetic condition with an estimated incidence of 1 in 35,000 births and is considered irreversible (Summar et al., 2013). This disease necessitates lifelong pharmacological treatment. The deficiency of these enzymes, coupled with the accumulation of dietary protein and the ongoing function of the urea cycle, results in the buildup of ammonia in the body. Ammonia, though naturally present in the body, becomes neurotoxic and fatal when it accumulates. The urea cycle is essential for its removal, and any disruption in this process leads to harmful levels of ammonia in the body (Dasarathy et al., 2017). As a genetic condition, urea cycle disorders present an immediate concern for pediatric patients, typically diagnosed with the appearance of symptoms. Prompt treatment is essential to prevent permanent neurological damage and other severe complications. Adult onset of the condition is possible through any excess of ammonia/nitrogen-containing compound consumption or a disorder of metabolism, such as drug-induced hepatotoxicity (Shakerdi and Ryan, 2023).

**FIGURE 4:**
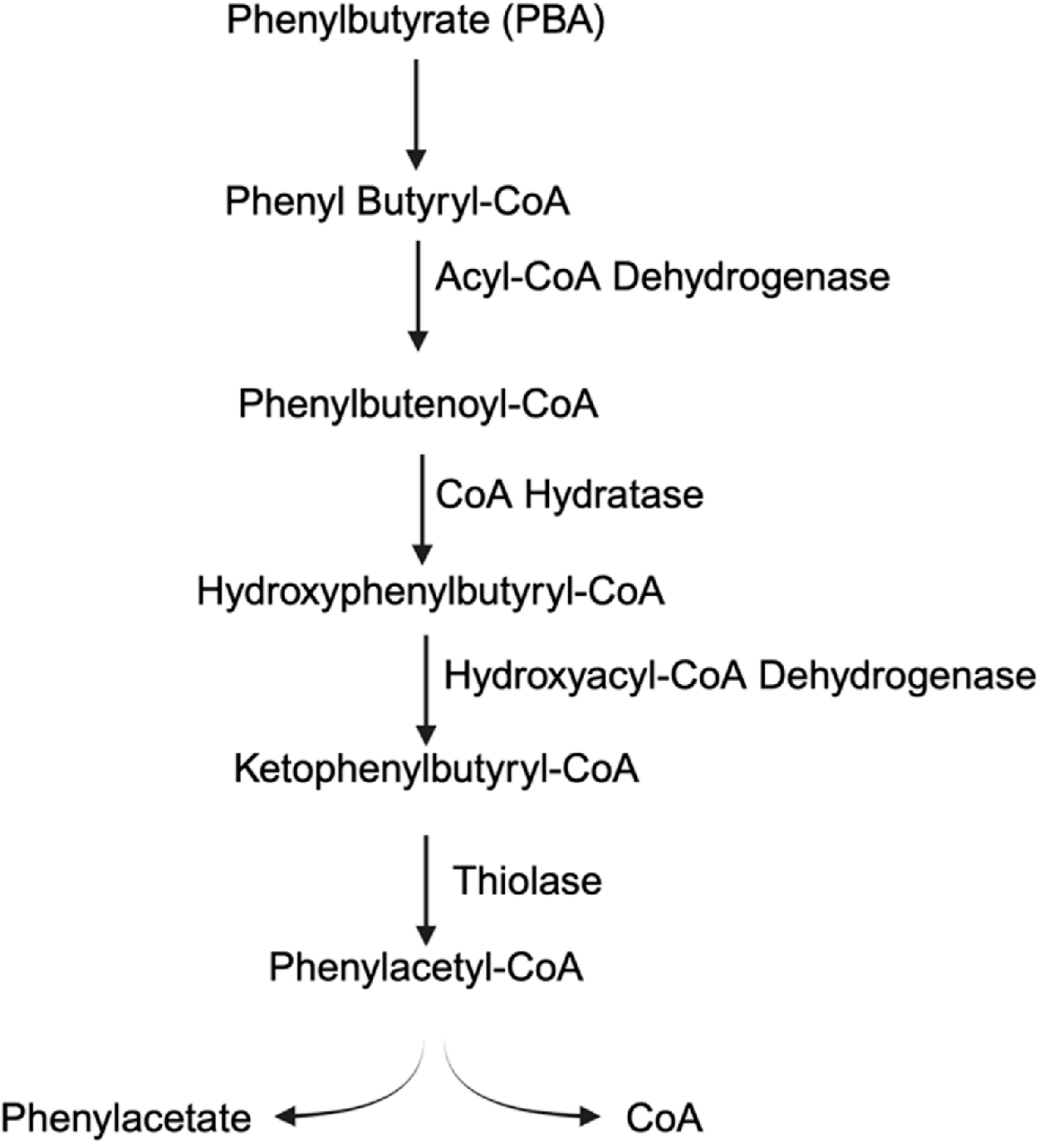
Conversion scheme of phenylbutyrate (PBA) to phenylacetate (PAA).

In certain cases, such as for the treatment of urea cycle disorders, 4-PBA is a prodrug that needs to be converted into phenylacetate (PAA) *in vivo* (Figure 4). Phenylbutyrate is converted into phenyl butyryl-CoA, which is then turned into phenylbutenoyl-CoA via acyl-CoA dehydrogenase (Kormanik et al., 2012). Phenylbutenoyl-CoA is converted into hydroxyphenylbutyryl-CoA by CoA hydratase, which is further converted into ketophenylbutyryl-CoA by hydroxyacyl-CoA dehydrogenase (Kusaczuk et al., 2015). Finally, ketophenylbutyryl-CoA is converted into phenylacetyl-CoA by the thiolase, and the CoA is hydrolyzed off to leave the active molecule phenylacetate (Kusaczuk et al., 2015). Phenylacetate is conjugated with glutamine into phenacetylglutamine via glutamine N-acyltransferase, a mechanism that allows for nitrogen scavenging. The removed (Misel et al., 2013). 4-PBA is currently the first-line treatment for the UCD and requires daily administration to aid in the removal of dietary glutamine. In adults, the drug is dosed 9.9–13 g/m^2^ per day in 3–6 equally divided doses, with a maximum dose of 20 g/day. In infants and children less than 20 kg, 4-PBA is dosed at 450–600 mg/kg/day in 3–6 divided meals/feedings with a maximum dose of 20 g/day through oral powder or pellets (Häberle et al., 2019).

## 4 Cardiovascular diseases

As the leading cause of death in the United States, cardiovascular disease (CVD) presents a significant need for further education and research (Benjamin et al., 2017). In 2020, estimates indicated that CVD was the cause of 19.05 million deaths globally, increasing by 18.71% since 2010 (Tsao et al., 2023). Major risk factors associated with CVD include hypertension, hypercholesterolemia, obesity, diabetes, and smoking (Lynn et al., 2019). While CVD continues to remain relevant, effective, and accessible treatments are being extensively researched to reduce the high mortality rate of CVD, with 4-PBA demonstrating promising results in alleviating associated manifestations and conditions of CVD through aiding in the immune response and serving as a chemical chaperone in ER stress (Fu et al., 2016; Lynn et al., 2019).

### 4.1 Phenylbutyrate and atherosclerosis

Atherosclerosis is characterized by the formation of cholesterol plaques along damaged areas of the arterial endothelium. As a result of endothelial dysfunction, lipoproteins accumulate, inducing the collection of macrophages that absorb lipids, creating “foam cells”. These clusters of cholesterol-rich macrophages are classified as early lesions (Lusis, 2000; Lynn et al., 2019). Macrophage apoptosis plays an important role in acute clinical complications of atherosclerosis, as it is known to create the necrotic cores that induce thrombosis, inflammation, and proteolytic plaque breakdown (Moore and Tabas, 2011). A recent study utilized chow-fed apolipoprotein (Apoe^−/−^) mice to isolate the effect of 4-PBA from proatherogenic factors linked to a high-fat diet. This report reveals that lesion growth was inhibited, suggesting that 4-PBA directly affects the macrophages that constitute atherosclerotic lesions (Lynn et al., 2019). The role of 4-PBA in the induction of heat shock protein 27, known to reduce lipid accumulation in macrophages, has been investigated (Kuang et al., 2017). Reports indicate that nuclearization of heat shock factor 1 (HSF1), a transcription factor for HSP27, increased following 4-PBA treatment in Apoe^−/−^ mice. Therefore, it has been hypothesized that 4-PBA inhibits lesion growth by inducing HSF1 nuclearization, a transcription factor of HSP27. Researchers theorize that HSF1 siRNA treatment hinders the protective cell attachment role of 4-PBA, independent of its antioxidant properties (Lynn et al., 2019).

### 4.2 Phenylbutyrate and adriamycin-induced cardiac injury

Adriamycin (ADR), also known as doxorubicin, is an anticancer drug that may induce cardiomyopathy and heart failure, limiting its clinical dosage (Octavia et al., 2012). ADR is known to induce oxidative stress, which is a side effect associated with cardiac injury (Chen et al., 2006). 4-PBA, on the other hand, has been shown to reduce oxidative stress (Ryu et al., 2005). Reports indicate that 4-PBA provides protection against cardiotoxicity in wild-type C57BL/6 mice. It was initially hypothesized that 4-PBA increases levels of manganese superoxide dismutase (MnSOD), an antioxidant enzyme, via its HDACI functionality to reduce oxidative stress. However, a more recent study shows that even in the absence of histone acetylation, 4-PBA maintains its protection against cardiotoxicity, suggesting that the underlying mechanism of 4-PBA is more associated with its ER chemical chaperone characteristics (Fu et al., 2016). Reports on the effect of 4-PBA on ADR-induced cardiac injury have delivered promising results, with some studies reporting a reduction in ultrastructural cardiac tissue damage by as much as 70%. The mechanisms underlying this quality remain unclear and require further research (Daosukho et al., 2007).

## 5 Hepatic disease

With around two million annual deaths, liver disease accounts for 1 in every 25 deaths worldwide (Devarbhavi et al., 2023). It is further estimated that 1.5 billion individuals have chronic liver disease worldwide (Moon et al., 2020). Major risk factors associated with the two most common hepatic diseases, non-alcoholic fatty liver disease (NAFLD) and alcoholic liver disease (ALD), include genetic susceptibility, insulin resistance, hypertension, obesity, type 2 diabetes, metabolic syndrome, and alcohol consumption (Lim and Bernstein, 2018; Hart et al., 2010). Left untreated, inflammation and tissue damage in chronic liver disease can progress to irreversible cirrhosis, with hepatocellular carcinoma (HCC) occurring in 80%–90% of those patients (Asafo-Agyei and Samant, 2023; Toshikuni et al., 2014). While being used clinically to treat urea cycle disorders (Kolb et al., 2015), 4-PBA’s role in the protein degradation process is a potential method for treating chronic liver disease and its manifestations (Chen et al., 2023).

### 5.1 Non-alcoholic fatty liver disease (NAFLD)

Driven by overnutrition, NAFLD is characterized by the accumulation of excessive lipid droplets and resulting lipotoxicity in liver cells (Powell et al., 2021). Macrophage infiltration of the expanded visceral adipose tissue compartment aids in the creation of a pro-inflammatory state promoting insulin resistance, with unsuitable lipolysis resulting in persistent fatty acid delivery to the liver (Friedman et al., 2018). The imbalance in lipid capacity subsequently leads to the formation of lipotoxic tissues contributing to oxidative stress, inflammation, apoptosis, and fibrogenesis (Sanyal, 2019). In a murine model, 4-PBA was demonstrated to resolve fatty liver disease with a decrease in liver triglyceride content and normalization of the liver functional enzymes alanine aminotransferase and aspartate with treatment, in addition to restoring systemic insulin sensitivity and enhancing insulin action in adipose tissues, liver, and muscle of mice with obesity-induced lipid accumulation in the liver (Özcan et al., 2006). 4-PBA was also found to play a protective role in lipid accumulation and lipotoxicity through the activation of autophagy by reducing lipid accumulation and apoptotic parameters *in vitro* in the Huh7 human hepatoma cell line (Nissar et al., 2017).

### 5.2 Rifampin-induced liver injury

Characterized by harm to hepatocytes and other hepatic cells, drug-induced liver injury is known to be a complex liver disease that can result in acute liver failure and death (Danan and Teschke, 2015; Andrade et al., 2019). Rifampin (RFP), a tuberculosis drug, has been known to induce liver injury such as hyperbilirubinemia and cholestasis (Capelle et al., 1972; Su et al., 2021). RFP-induced liver injury is associated with oxidative and ER stress as well as multidrug resistance-associated protein 2 (MRP2) (Bozaykut et al., 2016; Yew et al., 2018; Xu et al., 2020). A recent study reported that 4-PBA offers protective effects against RFP hepatotoxicity in L02 cells via the PERK-ATF4-CHOP signal pathway. The data suggest that 4-PBA inhibits the expression of PERK, ATF4, and CHOP, reducing ER stress in this cell line (Zhang et al., 2016). A later study indicates that 4-PBA treats RFP-induced liver injury by reducing endocytic retrieval of MRP2 and increasing MRP2 protein, reversing the effects of RFP. It was hypothesized that the increase in MRP2 protein inhibited the RPF degradation of GP78. Studies also report that 4-PBA treatment attenuates oxidative stress, as indicated by reduced Ca^2+^ levels after treatment (Fiskum and Pease, 1986; Chen et al., 2022). 4-PBA also reduces RFP-induced upregulation of various ER stress markers, confirming its chemical chaperone functionality (Chen et al., 2022). Moreover, 4-PBA has been shown to act as a host-directed therapy by targeting a central cellular stress axis within the immune system rather than the pathogen itself. This is evidenced by its bacteriostatic effect against *Mycobacterium tuberculosis*, which enhances macrophage responses (Coussens et al., 2015). With these propitious findings, 4-PBA has demonstrated potential as a therapeutic drug for treating RFP-induced liver injury. However, the limitations of only *in vitro* studies currently should be noted in the extrapolation to clinical outcomes, and future *in vivo* work is required prior to definitively concluding such beyond speculation.

## 6 Renal disease

A leading cause of mortality worldwide, chronic kidney disease (CKD) currently affects over 10% of the population or around 800 million individuals (Kovesdy, 2022). CKD is characterized by the presence of kidney damage markers (frequently assessed as an albumin-to-creatine ratio (ACR) greater than 30 mg/g) (Levey and Inker, 2022) or decreased kidney function (a glomerular filtration rate (GFR) of less than 60 mL/min per 1·73 m^2^) for at least 3 months (Webster et al., 2017). Individuals with CKD often carry a poor prognosis, with an increased risk for the development of cardiovascular mortality and infections on while on life-time dialysis (Rosenberg, 2022). Major risk factors associated with CKD include hypertension, obesity, smoking, and cardiovascular disease, in addition to diabetes mellitus and analgesic medications (Kazancioğlu, 2013). While a cure for CKD does not currently exist, the chemical chaperone 4-phenylbutyric acid (4-PBA) has been demonstrated to stabilize protein conformation and improve protein folding by inhibiting endoplasmic reticulum (ER) stress associated with the disease in a multitude of experimental *in vitro* and *in vivo* murine and rat models, suggesting strong evidence of translational value to future preclinical and clinical applications of 4-PBA in the context of renal disease in humans (Mohammed-Ali et al., 2015).

### 6.1 Diabetic nephropathy (DN)

Characterized by increased urinary albumin excretion and loss of renal function (Campbell et al., 2003), diabetic nephropathy (DN) is a common progenitor to CKD caused by diabetes mellitus (Varghese and Jialal, 2023). 4-PBA has been demonstrated to alleviate the effects and delay the progression of DN. Hyperglycemia associated with DN can lead to the activation of the associated receptor of advanced glycation end products (RAGE). RAGE triggers the synthesis of nuclear factor κB (NFκB) and the generation of reactive oxygen species (ROS), which have been linked to the unfolded protein response (UPR) and maintain kidney damage by hypertrophy, inflammation, angiogenesis, endothelial dysfunction, and extracellular matrix (ECM) production (Agarwal, 2021; Cao and Cooper, 2011). In *in vivo* animal studies, 4-PBA was demonstrated to reduce basement membrane thickening, mesangial cell proliferation, mesangial matrix accumulation, and urine excretion in diabetic nephropathy (Luo et al., 2010), in addition to preventing endoplasmic reticulum stress-induced podocyte apoptosis resulting from hyperglycemia (Cao et al., 2016). Further studies evaluated the fibrotic and inflammatory effect of proteins on tubular cells (Cravedi et al., 2012). Through calcium release-induced ER stress, proteinuria stimulates the overexpression of the secretory protein lipocalin 2, resulting in tubular apoptosis and renal lesions. 4-PBA was found to delay renal deterioration in proteinuric mice by inhibiting calcium release-induced ER stress in this pathway (El Karoui et al., 2016).

### 6.2 NSAID-induced kidney injury

When overused, nonsteroidal anti-inflammatory drugs (NSAIDs) inhibit cyclooxygenase (COX) enzymes, causing a reduction in prostaglandin synthesis and resultant renal ischemia, decline in glomerular hydraulic pressure, and acute kidney injury (AKI) (Luciano and Perazella, 2023). AKI is another common progenitor of CKD and is characterized by a sudden and oftentimes reversible reduction in kidney function measured by creatinine or decreased urine volume (Goyal et al., 2023). In the setting of NSAID-induced AKI, NSAIDs disrupt the compensatory vasodilation response of renal prostaglandins to vasoconstrictor hormones released by the body, resulting in acute deterioration of renal function (Dixit et al., 2010). Damage to specific nephron segments in AKI, leading to tubular damage, is also associated with ER stress and the resulting UPR (Gómez-Sierra et al., 2021). In a model of acute kidney injury, 4-PBA treatment prevented damage to the outer medullary stripe of the kidney and reduced ER stress upregulation and CHOP-induced apoptosis (Carlisle et al., 2014). A common outcome of renal ischemia and a component of AKI progression to CKD associated with the UPR is renal fibrosis. In post-ischemic kidneys, 4-PBA promoted renal recovery and suppressed tubulointerstitial injury as demonstrated by the reduction of tubular atrophy, renal fibrosis, and myofibroblast activation (Shu et al., 2018). Furthermore, inhibition of ER stress by 4-PBA reduced the activity of the main element of renal fibrosis: transforming growth factor-beta (TGF-β), in addition to the chemical chaperone mimicking an ER chaperone in the kidneys and significantly reducing GRP78, important in its role in activation of transmembrane ER stress sensors and pro-apoptotic CHOP expression in a rat unilateral ureteral obstruction (UUO) model of renal fibrosis (Liu et al., 2016). It remains important to note that immunological and physiological differences between different model species may influence immune responses to renal injury, recovery patterns, as well as therapeutic outcomes of 4-PBA treatment in NSAID-induced kidney injury.

## 7 Neurological disorders

Neurological disorders account for almost 10 million deaths annually and 349 million disability-adjusted life years (DALYs), the sum of years with disability and years of life lost (Feigin and Vos, 2018; Ding et al., 2022). Options to reduce their morbidity and mortality are critically needed. Reports associated with two disorders, HSV encephalitis and Parkinson’s disease (PD), have demonstrated signs of success with 4-PBA treatment.

### 7.1 HSV encephalitis

Herpes simplex virus (HSV-1), an alpha herpesvirus, is a neurotropic virus that causes a variety of neurological diseases and has been shown to be the dominant cause of multiple systemic infections, including encephalitis (Kapoor et al., 2024; Gagan et al., 2024). Further, HSV establishes latency in infected cells, enabling lifelong infection of the host (Sharma et al., 2022; Kapoor and Shukla, 2023; Heldwein and Krummenacher, 2008). The mechanism behind HSV entry into the central nervous system (CNS) is uncertain, but reports suggest two possible pathways of infection including retrograde transport via the trigeminal ganglion (TG) and the olfactory bulb (OB), both of which support a unilateral HSV emergence mechanism in the CNS (Patil et al., 2022a; Patil et al., 2022b; Yadavalli et al., 2023; Jennische et al., 2015).

HSV-1 is reported to disrupt the unfolded protein response (UPR) that is employed in the presence of ER stress to prevent apoptosis (Burnett et al., 2012; Johnston and McCormick, 2020). A transcription factor found in the ER called cyclic adenosine 3′,5′-monophosphate (cAMP) response element–binding protein 3 (CREB3) has been associated with HSV, as it has been shown to be capable of transcribing certain HSV genes (Ye, 2013). It was also revealed that CREB3 attenuates the expression of CCAAT enhancer-binding protein homologous protein (CHOP), a proapoptotic regulator. HSV is known to decrease CHOP expression (Yadavalli et al., 2020).

In a recent study using a murine model, 4-PBA in conjunction with other anti-HSV nucleoside analogs such as acyclovir (ACV) exhibited increased efficacy in treating HSV encephalitis. Using ACV at 10 mg/kg or 4-PBA at 400 mg/kg, only partial protection was observed with an average of 20% weight loss and 50% animal death, with over half exhibiting behavioral abnormalities. An amalgam of ACV (10 mg/kg) and 4-PBA (100 mg/kg) demonstrated even greater protective effects, including less than 10% weight loss, 0% animal death, and no residual behavioral deficits post-recovery (Yadavalli et al., 2020). Importantly, the study also indicates that the combination of 4-PBA and ACV is effective against ACV-resistant strains of HSV and reduces renal toxicity (Yildiz et al., 2012; Yadavalli et al., 2020).

**FIGURE 5:**
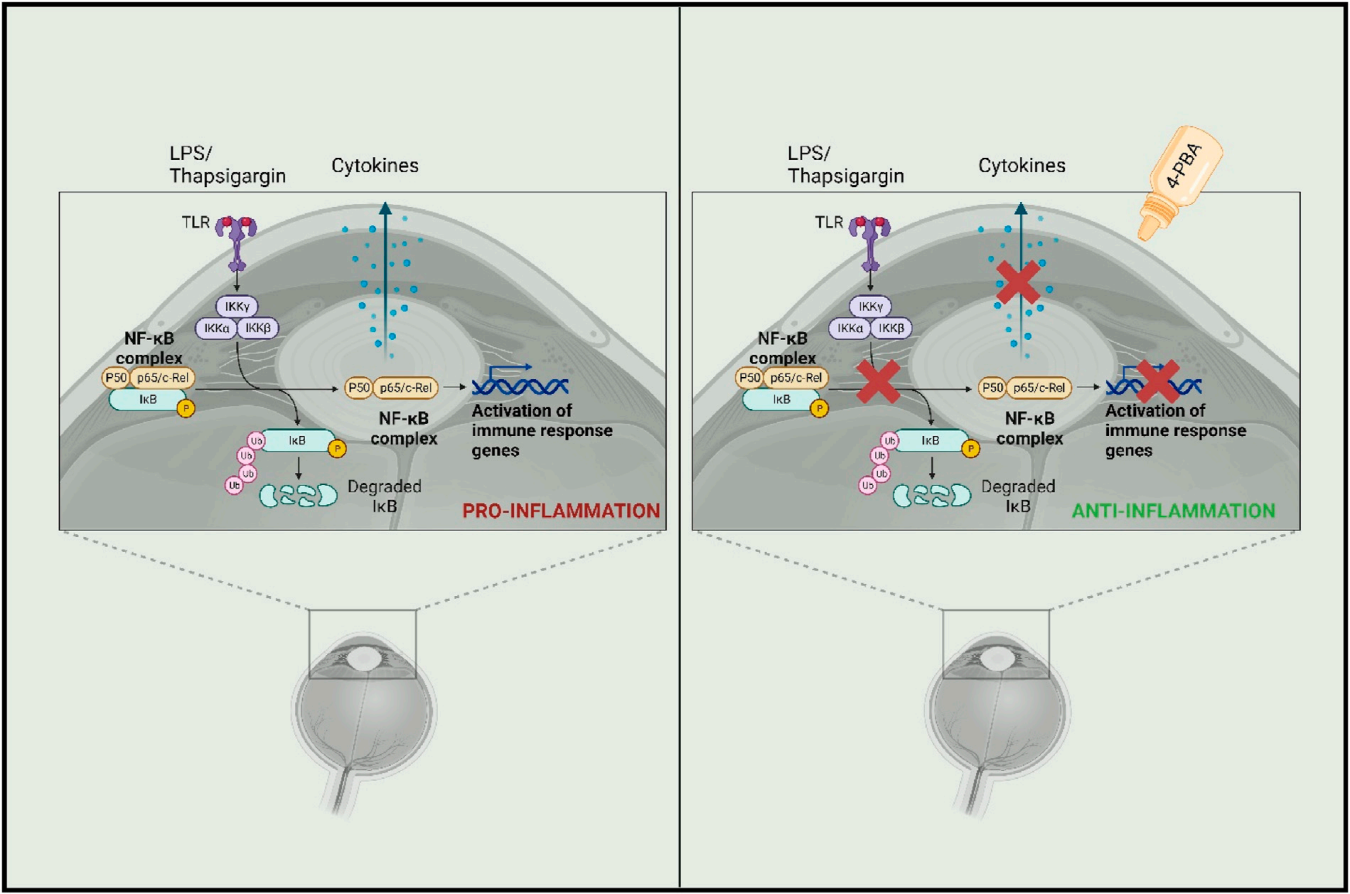
Mechanism showing potential anti-inflammatory activity of 4-PBA (Created in bioRender).

Mechanistically, the data indicate that 4-PBA silences CREB3 expression, increasing the nuclearization of CHOP and ATF4 (Yadavalli et al., 2020). 4-PBA has also been shown to inhibit proinflammatory molecules such as iNOS, TNF-α, and IL-1β in glial cells. It accomplishes this by utilizing p21ras, a small membrane protein that binds nucleotides, which suppresses transcription factor nuclear factor kappa B (NF-κB), the most integral transcription factor of the aforementioned proinflammatory molecules (Roy et al., 2012). Further scientific rigor is required to evaluate the full spectrum of safety and efficacy, but current results from murine models demonstrate the promise of 4-PBA in applications to HSV encephalitis (Figure 5).

### 7.2 Phenylbutyrate and Parkinson’s disease

As the second-most prevalent neurodegenerative disease in the United States, PD imposes a significant socioeconomic and quality-of-life burden on many patients (Surguchov, 2022). PD is a progressive motor neurodegenerative disorder that is characterized by the aggregation of misfolded α-synuclein of Lewy bodies and early death of dopaminergic neurons in the substantia nigra pars compacta (SNc) and striatum (STR) (Surguchov, 2015; 2022). Pathological markers of PD include excess dopamine and low levels of tyrosine hydroxylase (TH), an enzyme that regulates dopamine synthesis (Tiwari et al., 2022). This surplus of dopamine is oxidized, increasing stress in the substantia nigra (SN) (Wang and Michaelis, 2010). An association between PD and unfolded protein response (UPR) dysfunction has also been reported, but a causal relationship remains uncertain (Costa et al., 2020). PD has also been linked to mitochondrial dysfunction, resulting in reactive oxygen species (ROS) generation and oxidative stress (Santos and Cardoso, 2012; Millichap et al., 2021). PD primarily affects the midbrain dopamine neurons, resulting in the basal ganglia experiencing dopaminergic deafferentation (Alexander, 2004). Consequent motor complications include tremors, bradykinesia, and rigidity (Gelb et al., 1999).

A recent *in vivo* study in a rat model found that 4-PBA attenuates PD-induced physiological effects, including dopaminergic neuronal death and motor impairment, when dosed at 120 mg/kg intraperitoneally. The study suggests that 4-PBA prevents TH depletion and excessive dopamine synthesis in both SN and STR with PD. 4-PBA also reduced aggregation of misfolded α-synuclein, demonstrating its chemical chaperone activity. In observance of their behavior, the experimental models of PD exhibited significantly enhanced motor function and coordination (Tiwari et al., 2022). This study investigates the relationship between the ER and dysfunctional mitochondria, as it has been reported to play a role in PD pathogenesis and oxidative stress, but the effect of 4-PBA on this crosstalk requires further investigation (Sironi et al., 2020; Tiwari et al., 2022). While this relationship remains uncertain, the data suggest that 4-PBA decreases oxidative stress.

While the mechanism is not complete, another experimental *in vivo* study on a murine model confirms the antioxidant functionality of 4-PBA in glial cells through 200 mg/kg body weight/day dosing via gavage. NADPH oxidase is known to be a significant source of ROS generation (Roy et al., 2012). The data indicate that 4-PBA exhibits inhibitory effects on p21^rac^ activation, which is an essential protein subunit of the activated form of NADPH oxidase complex (Bokoch and Knaus, 2003; Roy et al., 2012). This suggests that 4-PBA attenuates oxidative stress in activated microglial cells as NADPH oxidase catalyzes superoxide synthesis. Neuroinflammation is another factor that 4-PBA has been shown to attenuate (Roy et al., 2012). The full effect of 4-PBA on PD requires further research.

### 7.3 Amyotrophic lateral sclerosis (ALS)

Amyotrophic Lateral Sclerosis (ALS) is a genetic degenerative neurological condition that leads to the loss of motor neuron control and eventually death (Ilieva et al., 2023). Sodium phenylbutyrate/taurursodiol is approved by the FDA for use in ALS patients to slow disease progression and extend life. The mechanism of action of this combination for ALS therapy is not entirely understood. However, it is theorized that the combination of taurursodiol, a bile acid, and 4-PBA abrogates the pathways for cell death in the mitochondria and endoplasmic reticulum in the neuronal cells, with taurursodiol also aiding in the penetration of the blood-brain barrier (Khalaf et al., 2022). The main clinical trial providing evidence for sodium phenylbutyrate/taurursodiol is the CENTAUR phase 2 randomized controlled trial (Paganoni et al., 2020). In this RCT, 3 g of sodium phenylbutyrate and 1 g of taurursodiol were administered daily for 3 weeks and then twice daily either orally or through a feeding tube for 24 weeks in total. At the conclusion, patients taking sodium phenylbutyrate/taurursodiol had a slower rate of functional decline per month than the placebo group as measured by the ALSFRS-R score (−3.54% of total symptomatic score compared to −3.03 in the 4-PBA group) in addition to decreased adverse effects (12% compared to 19%) (Paganoni et al., 2020). Additionally, adverse effects occurring at >2% frequency in the sodium phenylbutyrate/taurursodiol group remained primarily gastrointestinal, with abdominal discomfort, diarrhea, and nausea all associated with taurursodiol rather than 4-PBA. A follow-up study of the initial trial showed that, on average, the treatment leads to a life extension of 7 months (Paganoni et al., 2021).

This drug is currently marketed under the name Albrioza (Europe) and Relyvrio (North America) and was approved by the FDA in 2022. The formulation is a sachet of 3 g sodium PBA and 1 g of taurursodiol to be taken orally after dissolution in water. Prior to the use of 4-PBA for ALS, riluzole and edaravone were the only available treatments to alleviate the symptoms of the condition. Currently, none of these pharmacological treatments are curative for the condition. Treatment options for ALS are incredibly limited, and the condition has such a high fatality rate that any potential options for treatment should be considered. Concurrent use of 4-PBA with riluzole or edaravone has not been thoroughly investigated.

## 8 Ocular disease

The DALYs of blindness and vision loss account for a cumulative 22.56 million years, with ocular conditions and diseases affecting at least 2.2 billion individuals globally (Yang et al., 2021; Demmin and Silverstein, 2020). Herpes simplex virus (HSV) is estimated to cause a lifelong infection in 90% of the worldwide population (Wald and Corey, 2007; Karasneh et al., 2021; Patil et al., 2022a; 2024). HSV is primarily conferred through contact with herpetic lesions, mucosal secretions, or the skin of an infected individual, with HSV-1 as the leading cause of orofacial herpes (Malkin, 2002). Ocular HSV-1 infection occurs as primary or recurrent episodes, with keratitis being the most common ocular manifestation of the virus, with other manifestations including secondary glaucoma, acute retinal necrosis, and herpetic blepharitis, iridocyclitis, uveitis, retinitis, and conjunctivitis (Koganti et al., 2021). Among these, herpes simplex keratitis stands out as the second leading cause of blindness after cataracts (Allison et al., 2020). Both topical and systemic administration of 4-PBA has been demonstrated to alleviate symptoms associated with ocular diseases in human corneal cells, monocytes, and *in vivo* murine models.

### 8.1 Ocular HSV-1 pathogenesis and 4-PBA treatment

ER stress caused by viral infection is a common trigger of the UPR’s cellular pathway response (Sano and Reed, 2013). Despite the foreign nature of HSV in the body, the infection prevents cells from clearing the virus or initiating apoptosis by disarming the UPR (Burnett et al., 2012). Rather, the virus hijacks the host’s ER membrane proteins in order to disarm the UPR and produce its own viral proteins in addition to hijacking the host’s protein transcription factor nuclear factor kappa B (NFκB) pathway (Johnston and McCormick, 2019; Amici et al., 2006). Neovascularization, neolymphangiogenesis, opacification, and scarring of the cornea associated with ocular HSV-1 are ultimately due to chronic inflammation by factors such as the host response mediated by the protein transcription factor nuclear factor kappa B (NFκB) (Koujah et al., 2019).

**FIGURE 6:**
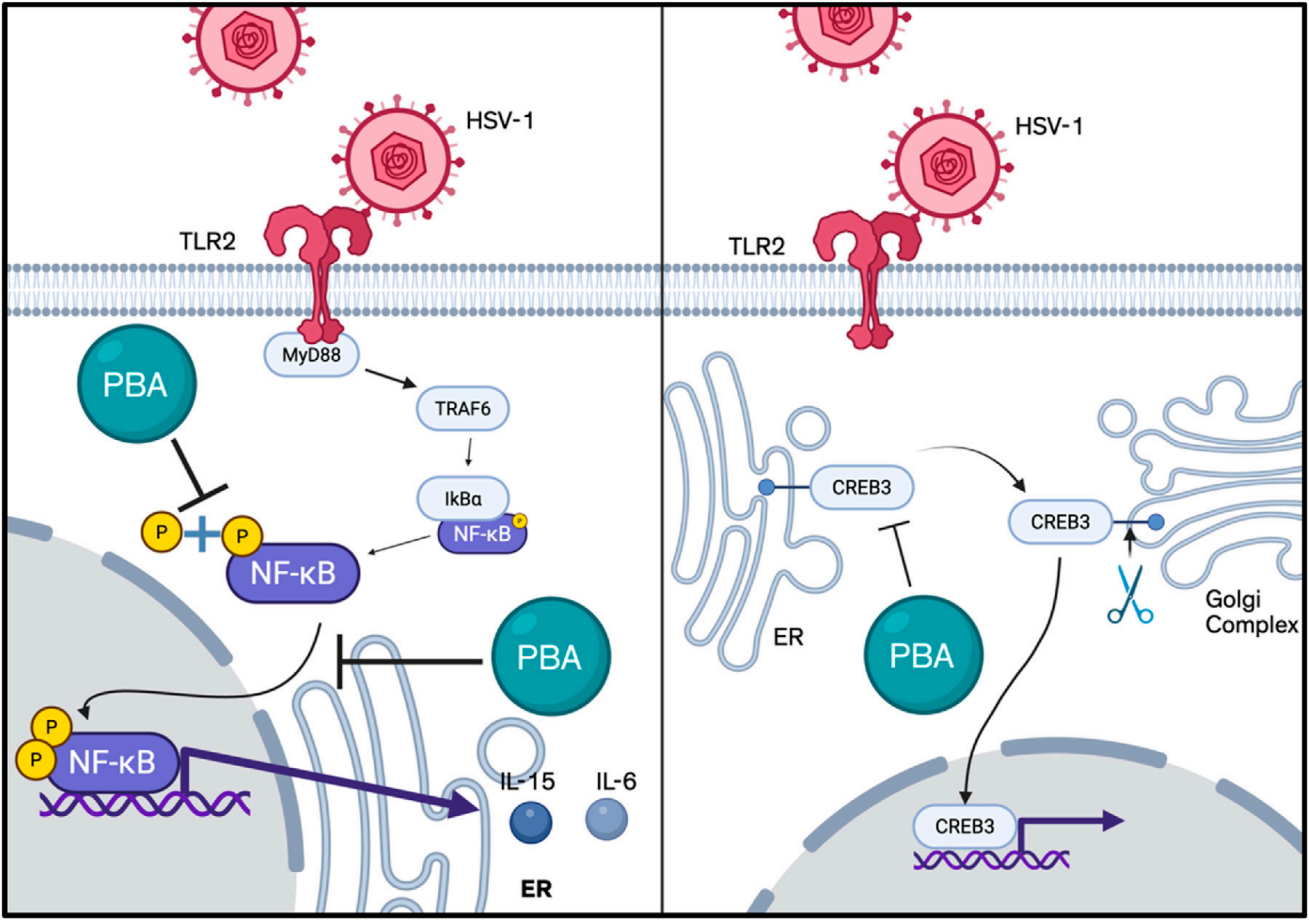
Known mechanisms of action of 4-PBA (Created in bioRender).

NFκB is upregulated in response to viral infection via the TLR2 and myeloid differentiation factor 88 (Myd88)/TN receptor-associated factor 6 (TRAF6) pathway and plays an important role in viral replication and in the upregulation of pro-inflammatory cytokines such as IL-15 and IL-6 (Takeda and Akira, 2004; Kurt-Jones et al., 2004). Accordingly, TLR2 knockout mice demonstrated significant resistance to disease expression and reduced levels of neovascularization and stromal inflammatory reactions at both early and peak times of viral response (Ahmad et al., 2008). Furthermore, phosphorylation of NF-κB p65 is associated with enhanced viral transcriptional activity (Hu et al., 2022). One of our studies found topical 4-PBA to inhibit the phosphorylation of p65 which enhances NF-κB transcriptional activity. In preclinical experimentation, 4-PBA was able to restrict the expression of pro-inflammatory cytokines potentially through the inhibition of NF-κB signaling, and facilitate the reduction of corneal thickness and ulceration in otherwise persistent herpetic keratitis, in addition to synergizing with existing corticosteroids such as dexamethasone to reduce inflammation (Figure 6) (Koganti et al., 2022).

The pro-viral transcription factor cyclic AMP-responsive element-binding protein 3 (CREB3), the homolog of HSV-1 VP16 required for the establishment of virus latency, resides within the ER, aiding the ER-associated degradation (ERAD) complex, binding to the host cell factor 1 to initiate transcription of HSV immediate-early genes, and playing a crucial role in HPSE-facilitated HSV-1 egress (Lu and Misra, 2000; Sampieri et al., 2019; Leitman et al., 2014; Yadavalli et al., 2020). One of our studies found CREB3, and its transcription and translation, to be the only protein upregulated among standard ER-resident regulators of ER stress including ATF6, PERK, eIF2A, CHOP, and calnexin during HSV-1 infection, with a decrease in viral load and higher cell death in HSV-infected cells with CREB3 silencing. The antiviral property of 4-PBA was demonstrated to mimic CREB3 silencing by inhibiting viral replication, preventing CREB3 expression, and increasing CHOP and ATF4 expression in the nucleus. Additionally, infected cells treated with 4-PBA did not demonstrate translocation of NFκB to the nucleus, preventing NFκB from inducing the transcription of inflammatory cytokines (Yadavalli et al., 2020).

### 8.2 Primary open-angle glaucoma (POAG)

Primary open-angle glaucoma (POAG) is a chronic, progressive, and irreversible condition accounting for 70% of all glaucoma cases (Quigley and Broman, 2006). POAG is characterized by increased resistance to drainage in the trabecular meshwork (TM), a filter-like tissue consisting of TM cells embedded within the extracellular matrix (ECM) that controls intraocular pressure (IOP) by regulating aqueous humor (AH) outflow (Abu-Hassan et al., 2014). In POAG, this blockage occurs regardless of the drainage angle between the cornea and iris remaining open, and intraocular pressure (IOP) gradually increases, resulting in damage to the optic nerve and progressive loss of peripheral vision followed by loss of central vision and subsequent blindness (Mahabadi et al., 2023; Kwon et al., 2009).

Of the current established treatment modalities for glaucoma in humans, topical eye drops are the initial mainstay of treatment along with selective laser trabeculoplasty (Garg and Gazzard, 2020). Given this, topical 4-PBA is likely the best route for administration in the control of POAG and may reduce the possibility for adverse effects associated with 4-PBA due to limiting systemic absorption. Systemic and topical administration of 4-PBA were found to be effective in reducing IOP in Tg-MYOCY437H mice, with twice-daily topical application resulting in IOP changes starting at 1 week. Mutations in the myocilin gene (MYOC) are the most common known genetic cause of POAG (Stone et al., 1997), and topical ocular 4-PBA was demonstrated to reduce phenotypes of glaucoma in mice with this mutation by reducing myocilin accumulation and ER stress in the TM, in addition to systemic administration of 4-PBA preventing glaucoma in the same mouse model altogether by promoting the secretion of mutant myocilin in AH and decreasing intracellular accumulation of myocilin in the ER to prevent TM cell death (Zode et al., 2011; 2012). Furthermore, ocular hypertension (OHT) is a major risk factor for the development of POAG due to elevated IOP (Vass et al., 2007). Topical ocular eye drops of 4-PBA were demonstrated to significantly lower IOP in mice with glucocorticoid (GC)-induced-OHT by preventing the synthesis and deposition of GC-induced ECM in TM, and showcased another ability of 4-PBA to degrade existing abnormal ECM in normal human TM cells/tissues through matrix metalloproteinase-9 (MMP9) expression (Maddineni et al., 2021).

## 9 Skin disease

As the body’s largest organ, the skin is implicated in a variety of associated conditions, diseases, and disorders. Skin and subcutaneous diseases are estimated to affect approximately one-third of the global population as the fourth most common type of human disease (Flohr and Hay, 2021). Prominent long-term alterations and stigma are further associated with many skin and subcutaneous diseases even after resolution, influencing the mental health and quality of life of patients in addition to physical health (Christensen and Jafferany, 2023; Wan et al., 2020; Yew et al., 2020; Yakupu et al., 2023). Additionally, skin and subcutaneous disorders place a high burden on national healthcare systems, with an estimated direct healthcare cost of $75 billion in the United States (Lim et al., 2017).

### 9.1 Melanoma

Skin cancers remain the most commonly diagnosed group of cancers, with malignant melanoma as one of the most rapidly increasing types of cancer worldwide (Arnold et al., 2022). The superficial spreading form of melanoma accounts for 70% of all cases, while other forms include nodular melanoma implicated in 15%–30% of cases, and lentigo maligna and acral lentiginous melanomas involved in <10% of cases (Liu and Sheikh, 2014). Accounting for approximately 5% of all cancer cases, melanoma remains implicated in over 80% of skin cancer-related deaths (Saginala et al., 2021). While primary cutaneous melanoma can often be managed by surgery, advanced metastatic melanoma is estimated to cause 57,000 annual deaths globally (Lopes et al., 2022).

Approximately half of all melanoma patients harbor mutations of the proto-oncogene BRAF encoding a serine/threonine protein kinase in the RAS-RAF-MEK-ERK kinase pathway, which in turn promotes cell growth and proliferation (Holderfield et al., 2014). The presence of BRAF mutations in most benign nevi, however, suggests additional features such as the UPR may serve as an enabling characteristic for the development of melanoma toward metastasis, as three effector pathways of the UPR (ATF6, PERK, and IRE1) have demonstrated increased activity in metastatic cells in comparison to non-metastatic cells (Kong et al., 2022; Sykes et al., 2016). Furthermore, the UPR serves as an inducer of fibroblast growth factor (FGF) expression and cell migration, with FCF/FGCR signaling contributing to intratumoral angiogenesis, melanoma cell survival, and therapeutic resistance (Czyz, 2019). While 4-PBA *in-vitro* was found to downregulate FGF1 and FGF2 genes generally upregulated in metastatic melanoma through reduction of the UPR and fibroblast growth factors in both metastatic and non-metastatic melanoma cell lines when dosed at 1 mM and 5 mM for 48 h, *in-vivo* treatment with the drug in this context has yet to be studied, but is speculated to require further modifications due to insufficient drug delivery, low potency, or metabolization as demonstrated by failure to reduce UPR activity in mice xenografted with both cell lines (Eigner et al., 2017).

### 9.2 Wound healing

While 4-PBA may not yet serve as an ideal candidate for the treatment of melanoma, the drug has demonstrated other dermatological applications such as aiding in wound healing. Elevated ER stress has been implicated in the impairment of chronic wound healing associated with chronic diabetic ulcers, pressure ulcers, and venous ulcers, with 4-PBA reducing ischemia-reperfusion injury through inhibiting apoptosis and decreasing the expression of ER stress markers in rat skin flap models, in addition to the drug improving keratinocyte migration in an *ex-vivo* model of venous ulcers from human leg tissue and improving the reepithelialization rate in a full-thickness human skin wound healing model through further reduction of ER stress markers (Schürmann et al., 2014; Cui et al., 2016; Yue et al., 2016; Bachar-Wikstrom et al., 2020). Furthermore, our previous study on 4-PBA mimicking CREB3 silencing found 4-PBA to be effective in controlling HSV-1 skin infections through the drug demonstrating viral inhibition in human skin graft models of HSV-1 when 10 mM of 4-PBA was administered systemically (Yadavalli et al., 2020).

## 10 Limitations of phenylbutyrate

Whilst 4-PBA shows good efficacy for a number of conditions and through aiding in host immune response, inhibiting ER stress, and promoting healing from a variety of injuries, there are a number of identified adverse effects associated with the use of 4-PBA. There are two major types of limitations associated with 4-PBA, being usage limitations and formula limitations.

### 10.1 Usage limitations

One major and recurring adverse effect is seen in several clinical trials of 4-PBA gastrointestinal disturbance. Patients reported nausea, vomiting, and indigestion as a result of standard oral 4-PBA doses. Another common side effect is an unpleasent odor in patients’ urine and sweat. As treatment with sodium 4-PBA leads to an increase in the amino containing metabolites, these need to be excreted at a higher frequency and concentration than usual. This increase in the excretion of ammonia-containing compounds leads to an unpleasant smell for the patient’s bodily excretions. The most common side effect observed is irregular menstruation. It was found to occur in 23% of female patients. Whilst not a directly harmful side effect, it can be distressing for patients. As 4-PBA is marketed as a sodium salt and is required to be taken in a high dose, the elevated sodium levels in the patient often lead to additional complications. Around 10% of patients taking oral 4-PBA reported edema or hypernatremia. The doses taken for urea cycle disorders are much larger than any other dose with initial loading doses of 250 mg/kg (Häberle et al., 2012). Additionally, 4-PBA may be used cautiously in patients with kidney and/or liver disease using other medications. Given that systemically absorbed 4-PBA is metabolized jointly by the kidneys and liver (Kim et al., 2020), it is reasonable to assume that there may be drug-drug interactions with various renally excreted drugs, such as certain antibiotics, diuretics, and beta-blockers, as well as hepatically metabolized drugs such as NSAIDs and benzodiazepines.

Many of these side effects are dose-dependent due to 4-PBA and its metabolites contributing to body processes. Treatment for urea cycle disorders requires much higher doses than standard agnostic/antagonist drugs. Additionally, the entirety of the adverse effect reporting is for oral treatment with sodium 4-PBA. Further side effect studies and reporting will be required for possible 4-PBA repurposing.

### 10.2 Formulation limitations

PBA has a very salty and bitter taste and the palatability of 4-PBA has a major impact on patient adherence and compliance (Peña-Quintana et al., 2017; Cederbaum et al., 2023; Koren et al., 2016). As patients are already on a protein-restricted diet, taste modulation can further decrease adherence. Whilst not a harmful side effect, its effect on adherence, especially in pediatric patients is a major limitation for oral therapy.

Coating 4-PBA in polymers and sugars has shown some success in improving patient response and compliance. ACER-001 (a polymer coated sodium PBA oral formulation) is currently in clinical trials for urea cycle disorders. Prior improvements in the taste have been successfully approved such as PBA granules produced by Lucane Pharma and Horizon Pharma and the development of taste-masked PBA formulations will continue in the future. Conversion of PBA into a glyceride prodrug, glycerol PBA has been quite successful in improving patient acceptability and adherence. Glycerol PBA is a tasteless viscous liquid that is required to be taken with a lesser frequency compared to the sodium PBA formulations. However, the cost of the commercially available glycerol PBA oral solution (Ravicti) is quite high (Vara, 2019).

For the next-generation PBA formulations, it may be possible to take advantage of the low melting point of the acid form of the PBA (sodium-free) to develop sodium-free PBA formulations using techniques such as hot melt extrusion or melt emulsification.

## 11 Conclusion

**FIGURE 7:**
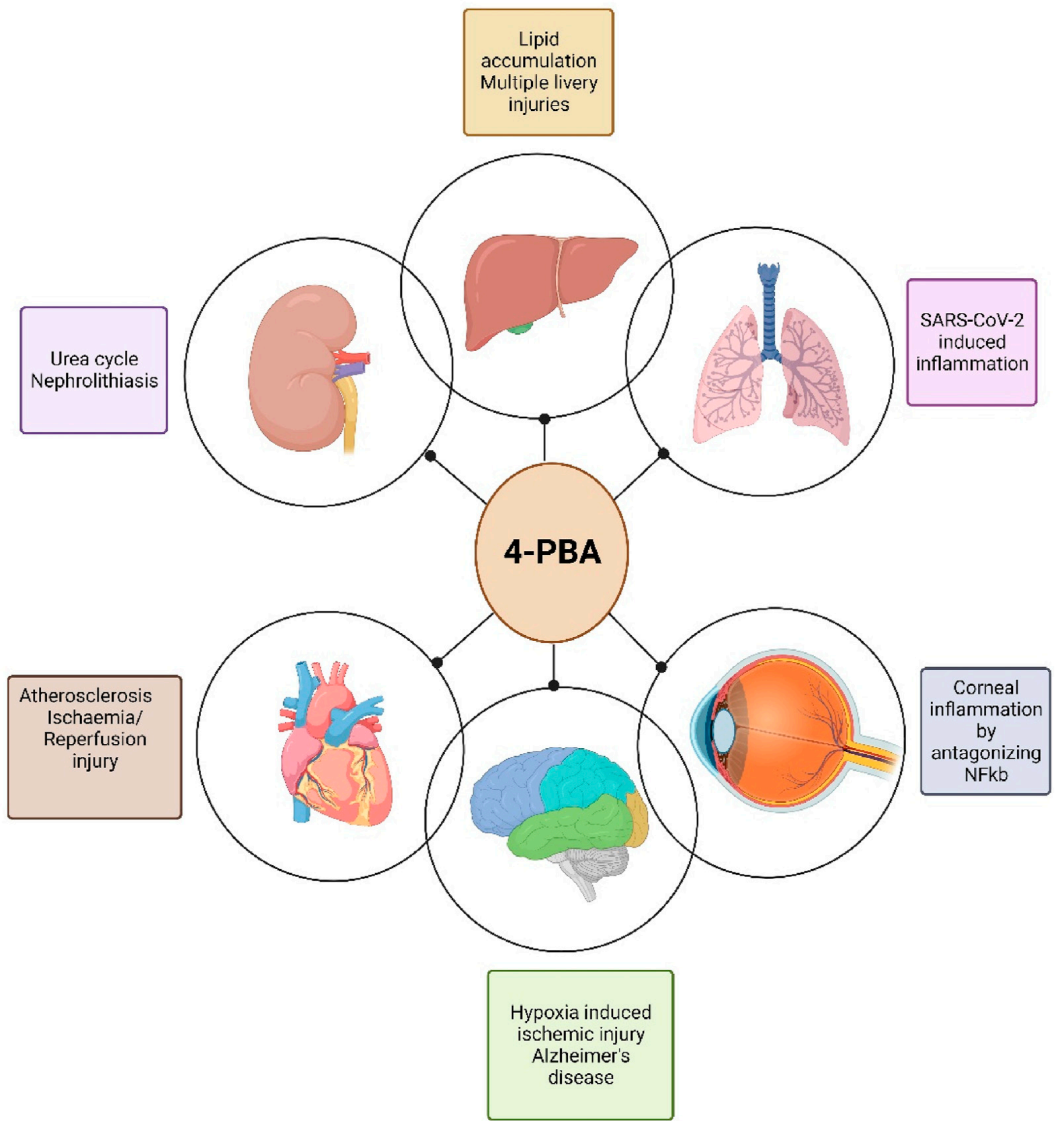
Representation of organs targeted by 4-PBA (created in bioRender).

While originally identified as a chaperone molecule with targets in the ER stress pathway of genetic disorders, 4-PBA has emerged as a promising drug to treat diseases in the cardiovascular, hepatic, renal, neurologic, and ocular systems, among others. While clinical evidence is currently only available for urea cycle disorders in ALS in the context of diseases discussed in this review, the characterization and exploration of 4-PBA has the potential to augment existing treatment models and establish novel therapeutic approaches. Given the estimated burden of disease in each organ system, the development of new small molecules such as 4-PBA may be instrumental in the next frontier of pharmaceutics (Figure 7). Since its discovery, 4-PBA has been studied in a variety of different organ systems with new insights uncovered each time.

Within the cardiovascular system, 4-PBA retains its previously-known chaperone-like functions but has also been linked to gene regulation via histone deacetylation (Daosukho et al., 2007; Erbay et al., 2009; Lynn et al., 2019). Meanwhile, within the hepatic system, 4-PBA has been implicated as an autophagy regulator, and in ocular studies, 4-PBA appears to play an anti-inflammatory role secondary to its ER-stress-related function (Nissar et al., 2017; Koganti et al., 2022). Given its small size, 4-PBA may interact with multiple cell pathways. Further research is thus needed to predict its molecular interplay with regulatory proteins in the transcription, ER-stress, and autophagic pathways, with *in silico* work being a particularly promising area of research.

Lipophilicity of 4-PBA, while an advantage for the creation of different formulations and routes of administration may also lend itself to deposition in fatty tissue (Basseri et al., 2009). The results of this could potentially lead to a longer than desired treatment window. Additionally, given that 4-PBA demonstrates its effects on a myriad assortment of organs, precautions should be made to prioritize organ selectivity to prevent off-target effects of employing this drug.

Since 4-PBA crosses the cell membrane with more ease than larger therapeutic agents, careful pharmacological tailoring is needed to make clinical applications while avoiding off-target effects. We have identified multiple organ interactions within our literature review, and a systemic release of the drug may cause unwarranted downstream consequences. For instance, 4-PBA was shown to promote hepatocellular carcinoma through PPAR-α and the migration of gastric cancer cells by the same histone deacetylation described earlier (Shi et al., 2020; Chen et al., 2021). Given its relative novelty, the tolerability of 4-PBA in different organ systems has yet to be ascertained, especially over a long treatment interval.

Each discovery regarding 4-PBA reveals its unique strengths compared to the standard line of care. Its small size presents an avenue for novel delivery methods and systemic administration for diseases of the cardiovascular, hepatic, renal, neurologic, ocular, and dermal systems. However, these applications require further research in order to shed insight into how 4-PBA may further augment our existing treatment models.
